# Strain-Controlled Low-Cycle Fatigue Behavior and Microstructure Evolution of the Hot-Work Die Steel at 700 °C

**DOI:** 10.3390/ma18245522

**Published:** 2025-12-09

**Authors:** Pengfei Jin, Lichao Shi, Chao Zhao, Cheng Zhang, Jinfeng Huang

**Affiliations:** 1 State Key Laboratory for Advanced Metals and Materials, University of Science and Technology Beijing, Beijing 100083, China; 2 Western Metal Materials Co., Ltd., Xi’an 710201, China; 3 School of Mechanical and Electrical Engineering, North University of China, Taiyuan 030051, China

**Keywords:** low-cycle fatigue, fatigue life, secondary cracking, carbide

## Abstract

**Highlights:**

**What are the main findings?**

- First systematic study of low-cycle fatigue behavior and microstructure evolution in novel 30Cr2Ni3MoWV hot-work die steel at 700 °C.
- The steel shows pronounced cyclic softening at high strain amplitudes (0.2–0.6%), leading to a steep reduction in fatigue life as strain increases.

**What are the implications of the main findings?**

- M23C6 precipitates, together with dislocation rearrangement and carbide coarsening, are major drivers of accelerated softening under elevated strain conditions.
- An Coffin–Manson model enables high-accuracy prediction of fatigue life for this steel at high temperature.

**Abstract:**

This study investigates the low-cycle fatigue behavior and microstructural evolution of a novel 30Cr2Ni3MoWV hot-work die steel at 700 °C under different strain amplitudes. High-temperature tensile tests demonstrated a tensile strength of 460 MPa and an elongation of 32%, confirming the material retains good ductility. Fracture analysis revealed ductile failure, supported by a 95% reduction in area. Low-cycle fatigue tests indicated notable cyclic softening at high strain amplitudes, with fatigue life declining rapidly as strain amplitude rose from 0.2% to 0.6%. A stress-softening coefficient model was established to describe this accelerated softening. Microstructural examination identified carbides (MC, M_7_C_3_, M_23_C_6_), which promoted secondary crack formation at 0.6% strain amplitude, contributing to early failure. TEM analysis further showed dislocation rearrangement, carbide coarsening, and martensite lath widening during cyclic loading. Among these, M_23_C_6_ precipitates were linked to increased softening at higher strains. The Coffin–Manson model parameters were optimized based on the relationship between fatigue life, plastic strain, and elastic strain. The model accurately predicted the steel’s fatigue life, with only a 0.01% deviation from experimental results. This work correlates accelerated softening and reduced fatigue life with three microstructural mechanisms—carbide coarsening, dislocation accumulation, and secondary cracking—offering valuable guidance for enhancing the high-temperature performance of hot-work die steels.

## 1. Introduction

Hot-work die steels are critical in manufacturing processes like die casting, forging, and extrusion, where they endure severe thermal–mechanical cycling [1,2,3]. Under such conditions, low-cycle fatigue resulting from accumulated plastic strain becomes a dominant failure mode, especially at high temperatures. This considerably shortens mold service life and impairs industrial productivity [4,5]. Improving the high-temperature low-cycle fatigue resistance of die steels is therefore essential to prolonging tool life, minimizing downtime, and enhancing process efficiency. As high-end manufacturing increasingly demands molds with better heat resistance and toughness, it is imperative to develop new high-alloy hot-work die steels and systematically investigate their high-temperature fatigue behavior. The newly developed 30Cr2Ni3MoWV steel, a high-strength and tough hot-work die steel, exhibits improved high-temperature strength and tempering softening resistance owing to the combined addition of carbide-forming elements such as Cr, Mo, W, and V. Nevertheless, studies on its low-cycle fatigue behavior, microstructural evolution, and the correlation between microstructure and fatigue properties at elevated temperatures remain limited, which constrains its wider engineering application.

Substantial research has been devoted to the low-cycle fatigue behavior of conventional hot-work die steels like H13 [6,7]. These studies establish that cyclic softening/hardening, carbide precipitation, and dislocation evolution critically affect high-temperature fatigue life [8,9,10]. Alloying elements such as W, Mo, and V improve strength via solid-solution strengthening and carbide formation; however, they can also promote crack initiation at incoherent interfaces [11]. Although empirical models, including the Coffin–Manson relation, have been developed for fatigue life prediction [12,13], they frequently fail to account for microstructural interactions under different strain amplitudes. Furthermore, while prior work has largely focused on room-temperature fatigue or monotonic tensile properties, the high-temperature low-cycle fatigue mechanisms of newer steels such as 30Cr2Ni3MoWV remain poorly understood.

This study systematically investigates tensile strength, fatigue life, and cyclic softening behavior of hot-work die steel (30Cr2Ni3MoWV) at 700 °C under different strain amplitudes. The microstructural evolution of fatigue fracture was analyzed using Scanning Electron Microscopy (SEM) and Transmission Electron Microscopy (TEM), explaining the influence mechanism of carbides, secondary cracks, and dislocations on fatigue behavior. Furthermore, the fatigue life prediction model has been optimized to provide high-precision prediction of steel fatigue life at elevated temperatures.

## 2. Materials and Methods

### 2.1. Experimental Materials

This study employed 30Cr2Ni3MoWV steel, which was prepared by vacuum induction melting and subsequent electro-slag remelting under a protective atmosphere. Its chemical composition is listed in Table 1. Samples were sectioned from the ingot into Φ110 mm × 110 mm rods via electrical discharge machining for subsequent heat treatment.

**Figure 1:**
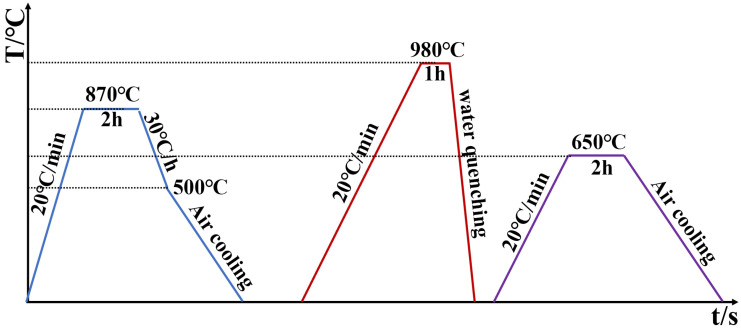
Heat treatment regime for the hot-work die steel.

The heat treatment comprised three stages. First, the specimen was heated to 870 °C, held for 2 h, furnace-cooled at 30 °C/h to 500 °C, and finally air-cooled to room temperature. Second, it was reheated to 980 °C, held for 1 h, and then water-quenched. Finally, the specimen was tempered at 650 °C for 2 h. A schematic of the complete heat treatment procedure is provided in Figure 1.

**Figure 2:**
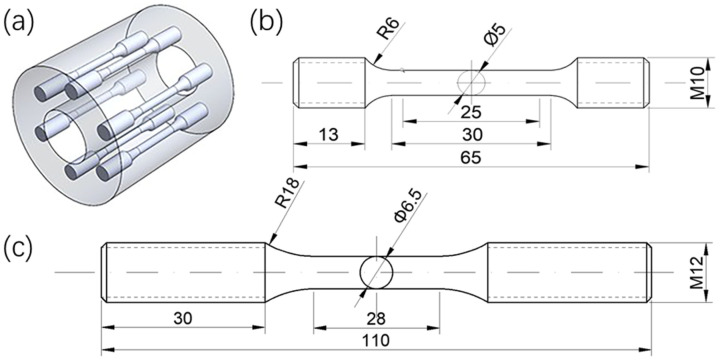
Sample processing diagram, (**a**) Schematic diagram of cutting position, (**b**) High-temperature tensile specimen, (**c**) High-temperature low-cycle fatigue specimen. All dimensions in the figure are in millimeters (mm).

The heat-treated samples (Figure 2a) were subsequently cut into an high-temperature tensile specimen based on the ASTM E21-2009 standard [14] (Figure 2b), and a high-temperature low-cycle fatigue specimen following the ASTM E606/E606M-2012 standard [15] (Figure 2c).

### 2.2. Experimental Methods

High-temperature tensile tests were conducted using an electronic universal testing machine (CIMACH, DDL50, Changchun, China). Prior to testing, the tensile specimens were heated to 700 °C and held for 10 min. The tensile strain rate was set to 2 mm/min, in accordance with the ASTM E21-2009 standard.

**Figure 3:**
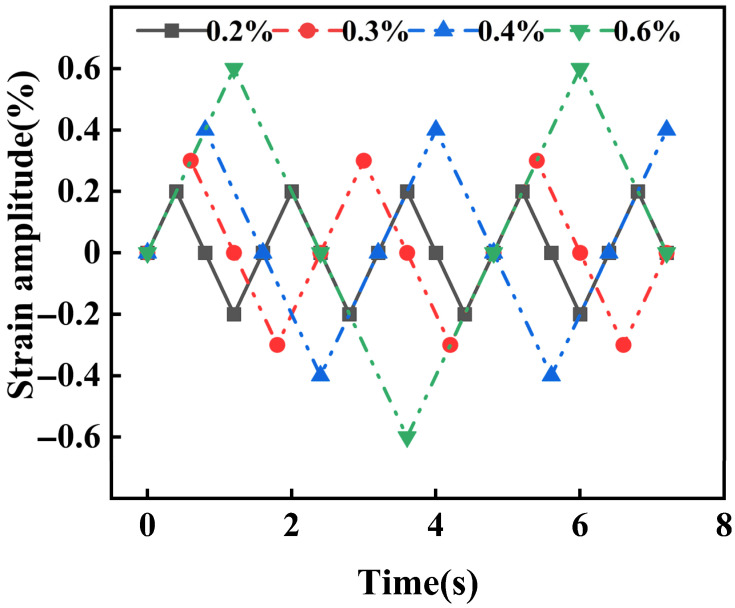
Continuous low-cycle fatigue cycling scheme diagram under different strain amplitudes.

High-temperature low-cycle fatigue tests were performed on an electronic hydraulic servo fatigue testing machine (MTS, NEW 810, Eden Prairie, MN, USA) in accordance with ASTM E606/E606M-2012. The tests were conducted at 700 °C under strain amplitudes of ±0.2%, ±0.3%, ±0.4%, and ±0.6% until specimen fracture (Figure 3). A triangular waveform was applied at a constant strain rate of 0.5%/s, with the strain controlled by a 25 mm extensometer. All tests maintained a mean stress of zero with a strain ratio (R) of −1.

### 2.3. Characterization

To investigate the high-temperature low-cycle fatigue behavior of the novel hot-work die steel, sectioned 30Cr2Ni3MoWV specimens were prepared for analysis. The samples were mechanically ground using SiC sandpaper (grades 400, 800, 1500, and 2000 mesh), polished with diamond suspension, and etched in a solution of HF:HNO_3_:H_2_O (1:3:6 by volume) for microstructural observation. Microstructure and carbide distribution were examined using an SEM (Zeiss, SUPRATM 55, Oberkochen, Germany) in back-scattered electron (BSE) mode. TEM specimens were prepared by electric discharge machining, followed by mechanical grinding with 800 to 3000 mesh sandpaper and electrolytic thinning using a dual spray apparatus (LEBO, TJ100-SE, Jiangyin, China). The electrolyte consisted of HClO_4_:C_2_H_5_OH (7:93 by volume) at −25 °C and 15 V to achieve an electron-transparent region of approximately 100 nm. Carbide morphology and microstructure were further analyzed using a transmission electron microscope (FEI, Tecnai F30, Hillsboro, OR, USA) operated at 300 kV, equipped with an energy-dispersive X-ray spectroscopy (EDS) system.

## 3. Results and Discussion

### 3.1. High Temperature Tensile Properties

**Figure 4:**
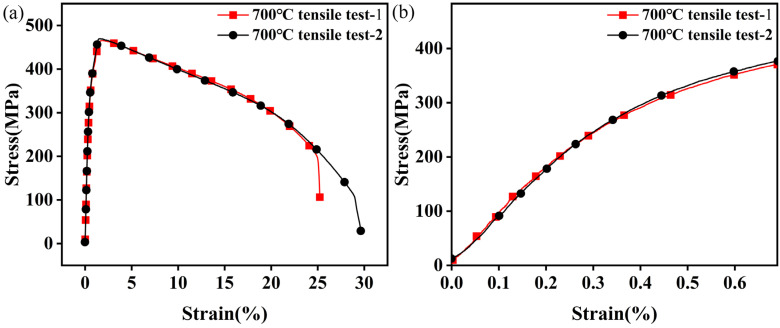
The engineering stress–strain curves. (**a**) High temperature tensile strength of 30Cr2Ni3MoWV steel at 700 °C, (**b**) is an enlarged view of strain from 0% to 0.7%.

Figure 4 presents the engineering stress–strain curves of 30Cr2Ni3MoWV steel tested at 700 °C and a strain rate of 0.5%/s. As shown in Figure 4a, the material initially displays linear elastic behavior with a modulus of 300 MPa, followed by continuous strain hardening until it reaches a peak tensile strength of 460 MPa at 1.34% strain. Beyond this point, stress decreases, marking the end of uniform elongation and the onset of localized plastic deformation. A significant stress drop near 25% strain indicates final fracture. Figure 4b provides a magnified view of the initial strain region from 0% to 0.7%. Four characteristic strain amplitudes—0.2% (yield initiation), 0.3% (early hardening), 0.4% (mid-stage hardening), and 0.6% (end-stage hardening)—were selected to study the low-cycle fatigue behavior. The 0.6% amplitude corresponds to a stress amplitude of 390 MPa, equivalent to 85% of the ultimate tensile strength. The close agreement between duplicate tests confirms good reproducibility, with minor deviations attributable to experimental variability.

**Figure 5:**
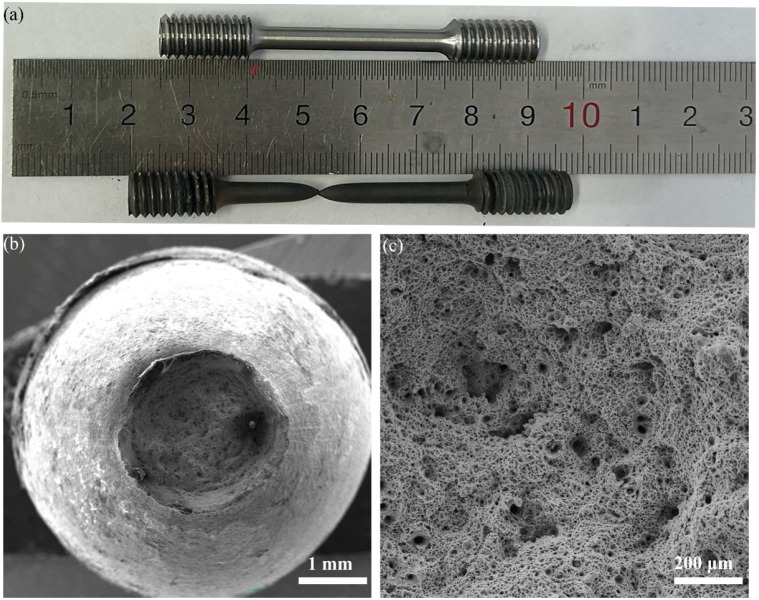
The fracture surface of the high-temperature tensile specimen. (**a**) Fractured specimen; (**b**) Overall fracture surface; (**c**) Dimple morphology at higher magnification.

The fracture measurement results are shown in Figure 5a. The gauge length increased from 25 mm to 33 mm, corresponding to a total elongation of 32%, which confirms retained ductility at high temperature. The fracture surface exhibits typical ductile characteristics (Figure 5b), with an area reduction of 95% and a cup–cone morphology in which the fibrous zone occupies approximately 65% of the total area. A magnified view of the dimple morphology is provided in Figure 5c. A comparison between the central and near-surface regions (within 1 μm of the edge) reveals a 30% reduction in dimple size near the surface, likely due to a constraint effect [16,17]. Overall, the fracture surface shows uniformly distributed dimples with considerable depth, indicative of extensive plastic deformation prior to fracture [18], consistent with the tensile curve in Figure 4.

### 3.2. Fatigue Analysis Under Different Amplitudes

**Figure 6:**
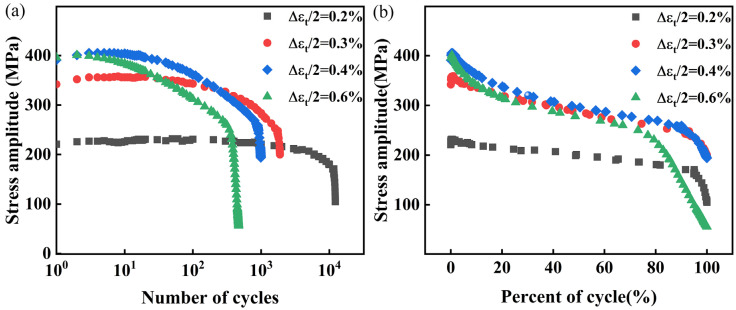
Stress amplitude curve of 30Cr2Ni3MoWV steel under different strain amplitudes. (**a**) number of cycles, (**b**) relative cycle.

The S-N curves depicting the peak stress behavior of 30Cr2Ni3MoWV steel at 700 °C under different strain amplitudes are presented in Figure 6. As the number of loading cycles increases, the stress of the material gradually decreases, indicating significant softening. As the strain amplitude increases from 0.2% to 0.6%, the softening of 30Cr2Ni3MoWV steel becomes more prominent, thereby accelerating the rate of stress reduction, as shown in Figure 6a. This observation suggests that higher strain amplitudes facilitate softening during fatigue, which is consistent with reports in the literature [19]. It is worth noting that at a strain amplitude of 0.2%, the material stress remains nearly constant as the number of cycles increases, indicating that 30Cr2Ni3MoWV steel has strong resistance to softening. When the number of cycles is converted to a relative number of cycles, as shown in Figure 6b, the entire curve can be divided into three distinct phases, i.e., the initial cycle stability stage, the intermediate softening stage, and the final transient fracture stage. In the initial stage of fatigue cycle loading, stress significantly increases, indicating cyclic hardening. In the intermediate stage, the stress reduction stabilizes, reflecting a steady fatigue behavior. In the later stage, it undergoes rapid softening, leading to a sharp decline in cyclic stress until fracture occurs.

**Figure 7:**
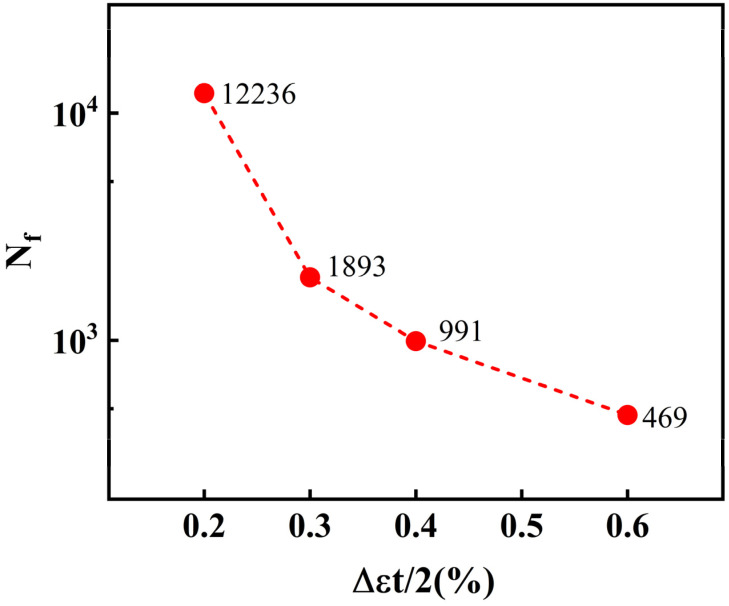
The fatigue life of 30Cr2Ni3MoWV steel.

The fatigue life of 30Cr2Ni3MoWV steel at 700 °C under different strain amplitudes is illustrated in Figure 7. At a strain amplitude of 0.2%, the material predominantly undergoes elastic deformation, with fatigue damage accumulating at a relatively slow rate. As the strain amplitude increases to 0.3%, the material transitions into the plastic deformation stage, where the internal microstructural defects (such as dislocations, microcracks, etc.) begin to rapidly propagate, resulting in a significant reduction in fatigue life. With a further increase in strain amplitude (from 0.3% to 0.6%), the material enters a pronounced cyclic plastic deformation state. In this stage, although the plastic strain is high, the rate of damage accumulation gradually slows down and tends to saturate. This widely observed phenomenon is mainly attributed to the development of stable dislocation cell/subgrain structures and the dynamic recovery process, which establish a balance between dislocation generation and annihilation, thereby inhibiting rapid crack initiation and propagation [20].

In order to characterize the stress changes more intuitively during the fatigue cycling process, the stress softening coefficient ($R_{\sigma }$) is used in this paper, and it is depicted in Equation (1) [21].

(1) $$R_{\sigma }=\frac{\Delta \sigma -\Delta \sigma_{0.5Nf}}{\Delta \sigma }$$

where Δσ is the stress amplitude, and $\Delta \sigma_{0.5Nf}$ corresponds to the stress amplitude at half of the fatigue life.

**Figure 8:**
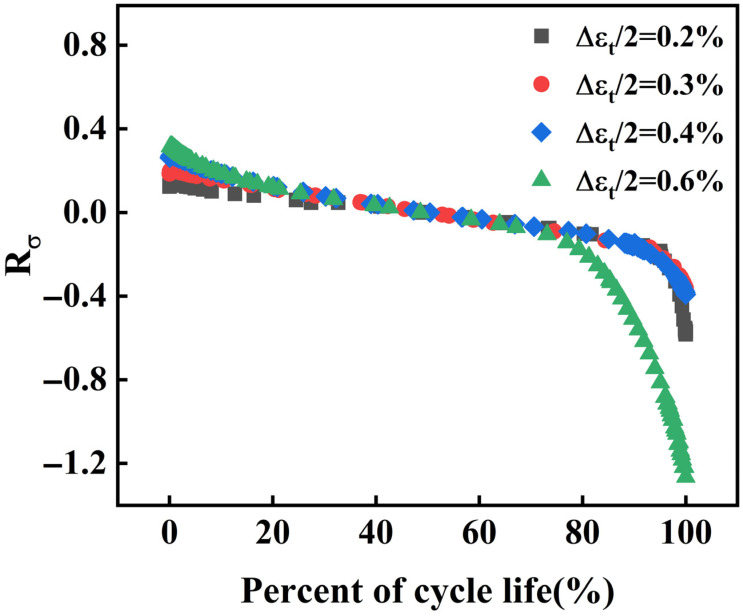
Stress softening coefficient under different strain amplitudes.

The relationship between $R_{\sigma }$ and $n/N_{f}$ is depicted in Figure 8. It exhibits a nonlinear trend, similar to the stress amplitude curves in Figure 6. The shape of the $R_{\sigma }$ curve is influenced by the progressive accumulation of fatigue damage. For strain amplitudes ranging from 0.2% to 0.4%, the $R_{\sigma }$ curves demonstrate a high degree of overlap. However, as the strain amplitude increases to 0.6%, obvious differences in the overlap of the curves are observed. This suggests that as the strain amplitude increases, the decreasing rate of $R_{\sigma }$ accelerates. Therefore, an increase in strain amplitude results in a more accelerated softening of the material, thereby detrimentally affecting its fatigue resistance.

**Figure 9:**
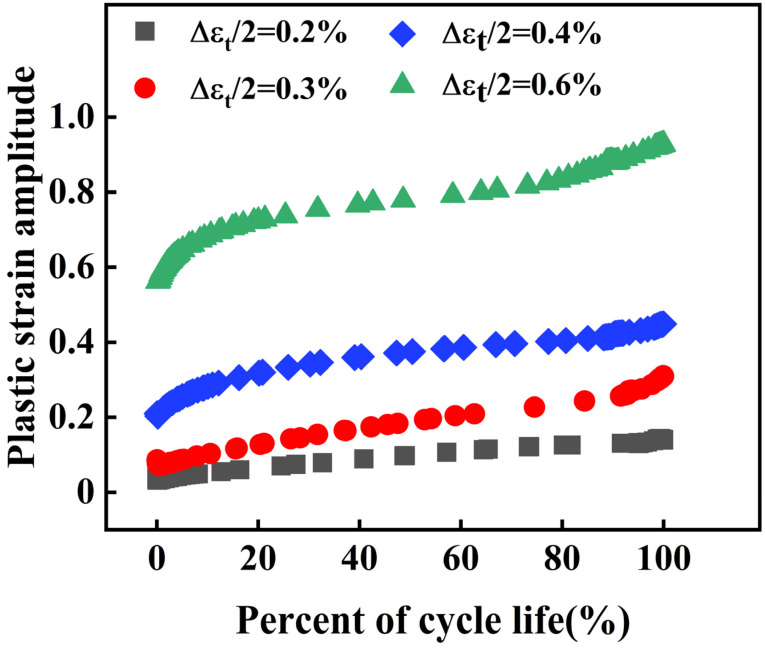
Plastic strain amplitude of 30Cr2Ni3MoWV steel under different strain amplitudes.

The low-cycle fatigue behavior of a material is predominantly influenced by the plastic strain [22,23]. The evolution of plastic strain during the fatigue loading process under different strain amplitudes is shown in Figure 9. For total strain amplitudes of 0.2% and 0.3%, the plastic strain amplitude is extremely low in the initial dozens of cycles and then continuously decreases to nearly zero with further cycling (Figure 9), indicating pronounced cyclic hardening of the 30Cr2Ni3MoVW steel during the early stage. When the percent of cycle life ($n/N_{f}$) is less than approximately 0.9, both the plastic strain amplitude (Figure 9) and stress amplitude (Figure 8) remain essentially stable. In the final ~10% of life, the stress amplitude drops rapidly, whereas the plastic strain amplitude increases sharply (Figure 9), signaling the onset of macroscopic crack propagation. This rapid increase in plastic strain amplitude in the tertiary stage is a direct consequence of cyclic softening induced by fatigue damage accumulation (microcrack initiation and growth, cavitation, etc.) rather than by recovery or recrystallization processes [24,25]. Furthermore, the plastic strain in the 30Cr2Ni3MoWV steel increases significantly with further fatigue loading cycles during the fatigue failure stage.

### 3.3. Fatigue Microstructure Analysis

**Figure 10:**
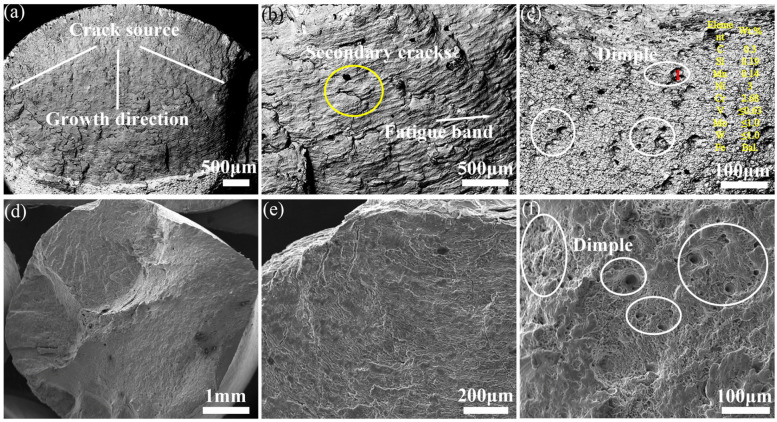
Morphology of low-cycle fatigue fracture expansion zone for 30Cr2Ni3MoWV steel, (**a**–**c**) strain amplitude is 0.6%, (**d**–**f**) strain amplitude is 0.2%.

The macroscopic morphology of fatigue fracture for 30Cr2Ni3MoWV steel under 0.6% strain amplitude is illustrated in Figure 10a–c. It includes three distinct regions, i.e., the fatigue crack origin zone, the fatigue crack propagation zone, and the instantaneous-fracture zone. It is observed from the fatigue crack origin zone that cracks at different heights continuously intersect during fatigue, resulting in the formation of steps that coalesce into small facets, as shown in Figure 10a. The main crack propagation surface exhibits river-like patterns aligned with the crack propagation direction, accompanied by perpendicular fatigue striations, as depicted in Figure 10b. Furthermore, secondary cracks are evident on the planes within the fatigue crack origin zone, originating from the crack tip, due to the strong concentration of stress. When the crack front encounters carbides or material property discontinuity induced by the incoherent interface between carbides and the matrix, secondary crack formation occurs at these carbide–matrix interfaces [26]. This significantly impairs the low-cycle fatigue performance of the material, especially at elevated temperatures [27]. Additionally, the spacing between fatigue striations is indicative of the material’s ductility and the propagation process of the fatigue crack. It is influenced by factors such as chemical composition, microstructure, and cyclic loading [28]. The wider fatigue striation spacing observed at a 0.6% strain amplitude suggests an accelerated crack propagation rate. It is found in the fatigue instantaneous-fracture zone that the fracture surface predominantly exhibits cleavage characteristics with river patterns, while dimples are rare and shallow (Figure 10c), indicating poor toughness. The EDS analysis of the cleavage facets in the instantaneous-fracture zone confirms the presence of carbide particles enriched in V and Cr. Previous studies have shown that tempered martensite undergoes significant recovery under external loading, generating dislocations that promote the diffusion of carbon and alloying elements [29]. Tempered martensite can experience significant recovery under external force, generating dislocations that promote the diffusion of carbon and alloying elements. Furthermore, the final overload fracture occurs in just a few microseconds. At such high strain rates, plastic deformation is essentially adiabatic, so nearly all plastic work is instantly converted into heat with no time for dissipation. As a result, the local temperature at the fracture surface rises by several hundred degrees Celsius above the test temperature, creating highly favorable conditions for rapid carbide growth and coarsening immediately ahead of and along the final fracture path. The mismatch in deformation and thermal expansion between the carbides and matrix gives rise to high stress fields around the sharp corners of irregular carbides, resulting in crack initiation [30,31,32]. Furthermore, when the main crack approaches these irregular M_7_C_3_ and M_23_C_6_ carbides, the interaction between the crack tip stress field and the stress concentration zones around the carbides significantly accelerates crack propagation [33], eventually leading to final catastrophic fracture.

At a strain amplitude of 0.2%, as shown in Figure 10d–f, the formation of fatigue crack initiation sites and fatigue bands is not distinctly observable. The size of the facets on the fatigue fracture surface decreases, and their distribution becomes increasingly dense. The reason is that the intensified interaction between microscopic slip bands, dislocation structures, and carbides within the material [34] results in the carbides exhibiting highly irregular morphologies with sharp corners, as seen in Figure 10d,e, which diminishes their coherent relationship with the matrix and generates intense stress concentration at carbide/matrix interfaces. It is observed from the fatigue instantaneous-fracture zone that a large number of deep dimples exist on the fracture surface, suggesting favorable toughness, as illustrated in Figure 10f. Consequently, the increase in the number of creep cavities and secondary cracks is the main reason for the sharp decrease in the high-temperature fatigue life of the material.

**Figure 11:**
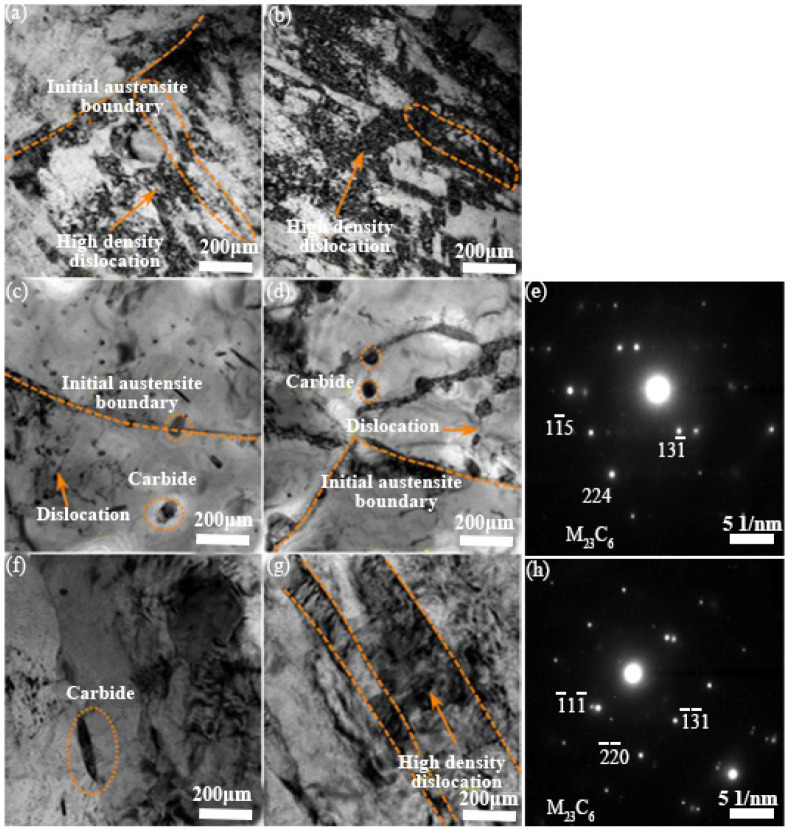
TEM micrographs of 30Cr2Ni3MoWV steel: (**a**,**b**) as-received condition with high-density dislocations (dashed box in (**b**)); (**c**–**e**) after fatigue at 0.2% strain amplitude; (**f**–**h**) after fatigue at 0.6% strain amplitude. (**e**,**h**) are the corresponding SAED patterns identifying M_23_C_6_ precipitates.

The TEM micrographs of 30Cr2Ni3MoWV steel, both prior to and following fatigue testing, are depicted in Figure 11. The initial lath martensite structure before fatigue testing, with dislocation tangles and cells inside the laths, is exhibited in Figure 11a,b. After fatigue, at a strain amplitude of 0.2%, as shown in Figure 11c,d, there is a significant alteration of the microstructure, with the dissolution of laths and dislocation cells, while precipitates emerge within the grains and at grain boundaries. According to the corresponding selected area electron diffraction (SAED) pattern (Figure 11h), these precipitates are still identified as the M_23_C_6_ phase. Early in cycling, dislocation rearrangement and annihilation predominantly contribute to material softening. As cycling continues, carbide precipitation and coarsening, influenced by both the number of cycles and temperature effects, take over, hindering dislocation motion [35,36]. At a strain amplitude of 0.6% Figure 11f,g, the martensitic lath structure is still retained. The corresponding SAED pattern, as shown in Figure 11h, confirms that the precipitates remain M_23_C_6_. Compared with the precipitates observed at the lower strain amplitude of 0.2%, these M_23_C_6_ carbides are markedly coarser and exhibit significantly larger sizes.

During isothermal fatigue, mechanical loading accelerates carbide growth, facilitated by the enhanced carbon diffusion under load [37]. Additionally, an increase in the density of dislocation tangles is observed at the higher strain amplitude of 0.6% compared to 0.2%. This behavior aligns with reports indicating that fatigue promotes dislocation rearrangement and increases microstructural heterogeneity [37]. Therefore, at a strain amplitude of 0.6%, the fatigue life is reduced, attributable to the coarser carbides and the increased dislocation density.

### 3.4. Low Cycle Fatigue Life Prediction Models

The cyclic stress–strain behavior is commonly depicted by cyclic stress–strain curves, which can be mathematically expressed using Equation (2) [38].

(2) $$\Delta \sigma /2=K^{\prime }(\Delta \varepsilon_{p}/2{)}^{n^{\prime }}$$

Here, Δσ/2 and $\Delta \varepsilon_{p}/2$ are the cyclic stress amplitude and plastic strain amplitude, respectively [37,38]. They can be obtained from the hysteresis loop at half-life. K′ and n′ are the cyclic strength coefficient and cyclic strain hardening exponent, respectively.

**Figure 12:**
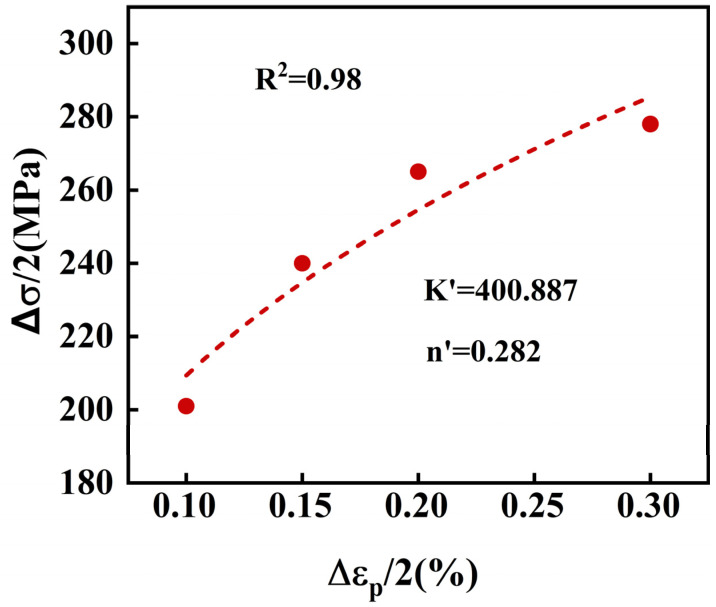
The fitting curve of cyclic stress–strain for 30Cr2Ni3MoWV steel.

The relationship between cyclic stress and strain is fitted using Equation (2) to determine the values of K′ and n′, as shown in Figure 12. The coefficient of determination (R^2^) is 0.98, and the values of K′ and n′ are 400.887 and 0.282, respectively.

By employing a double logarithmic coordinate system for linear regression analysis, the cyclic stress–strain relationship equation for 30Cr2Ni3MoWV steel can be derived as Equation (3).

(3) $$\Delta \sigma /2=400.887(\Delta \varepsilon_{p}/2{)}^{0.282}$$

**Figure 13:**
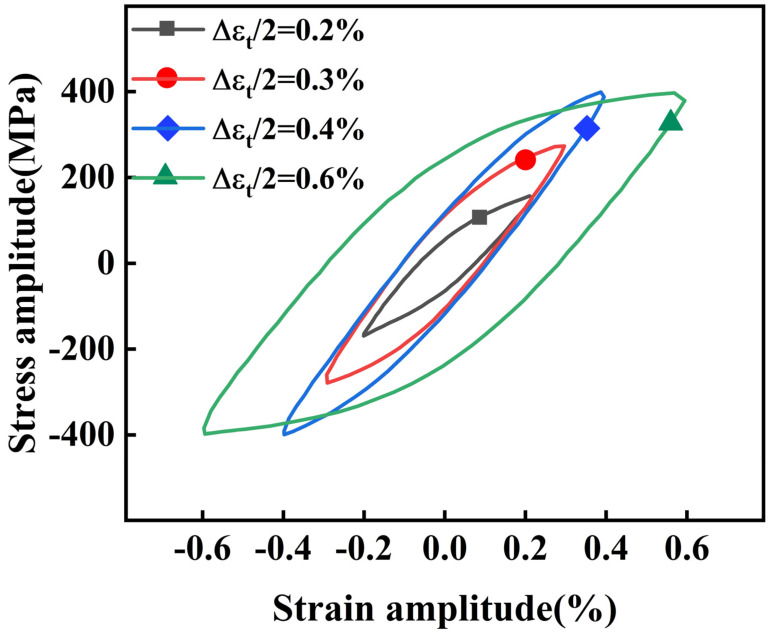
Half-life cycle hysteresis loop.

Based on the experimental data, the hysteresis loops of the material at different strain amplitudes and half-life cycle numbers were plotted, as shown in Figure 13. It is observed that the area of the hysteresis loop increases gradually as the strain amplitude increases. This indicates that the energy dissipated by the material’s plastic deformation increases with the rising strain amplitude, thereby compromising its cyclic toughness. Once the strain amplitude exceeds a critical threshold, plastic deformation dominates and the elastic component is negligible. The increased cyclic plastic deformation intensifies material softening, promotes microcrack initiation at stress concentration sites (such as carbide/matrix interfaces), and facilitates crack propagation. Consequently, the low-cycle fatigue life of 30Cr2Ni3MoWV steel is significantly reduced at higher strain amplitudes. Increased cyclic plastic strain energy dissipation has been widely recognized as one of the dominant damage mechanisms leading to a reduction in fatigue life [39].

**Figure 14:**
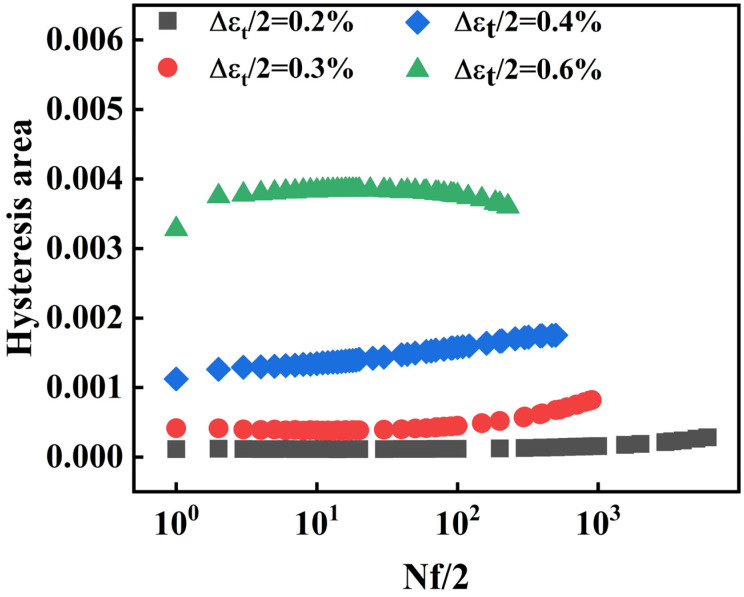
Relationship between fatigue cycles and hysteresis loop area.

The relationship between the number of cycles to half-life fatigue and the area of the hysteresis loop is shown in Figure 14. It is observed that the area of the hysteresis loop also increases gradually with increasing strain amplitude. At strain amplitudes of 0.2%, 0.3%, and 0.4%, the area of the hysteresis loop remains relatively stable during the initial fatigue loading. However, it subsequently increases gradually, demonstrating cyclic hardening. This excellent resistance to cyclic softening during fatigue is characteristic of 30Cr2Ni3MoWV steel**.** In contrast, at strain amplitudes of 0.6%, a distinct reduction in the hysteresis loop area is observed, signifying the onset of cyclic softening prior to reaching half-life.

The half-life hysteresis loop can be obtained from the Coffin–Manson relation given in Equation (4) [40,41].

(4) $$\frac{\Delta \varepsilon_{t}}{2}=\frac{\Delta \varepsilon_{e}}{2}+\frac{\Delta \varepsilon_{p}}{2}=\frac{\sigma_{f}^{\prime }}{E}(2N_{f}{)}^{b}+\varepsilon_{f}^{\prime }(2N_{f}{)}^{c}$$

where $\Delta \varepsilon_{t}/2$ is the total strain amplitude, $\varepsilon_{e}$ and $\varepsilon_{p}$ are the elastic and plastic strain amplitudes, respectively, and are obtained from the half-life hysteresis loop. $\sigma_{f}^{\prime }$ is the fatigue strength coefficient of the material. $\varepsilon_{f}^{\prime }$, b, c, and $N_{f}$ are the fatigue ductility coefficient, fatigue strength exponent, fatigue ductility exponent, and fatigue life of the material, respectively. According to the logarithmic law, Equation (4) is derived as Equations (5) and (6):

(5) $$\ln\frac{\Delta \varepsilon_{e}}{2}=b\ln\left(2N_{f}\right)+\ln\left(2\frac{\sigma_{f}^{\prime }}{E}\right)$$

(6) $$\ln\frac{{\Delta \varepsilon }_{p}}{2}=c\ln\left(2N_{f}\right)+\ln\left(2\varepsilon_{f}^{\prime }\right)$$

**Figure 15:**
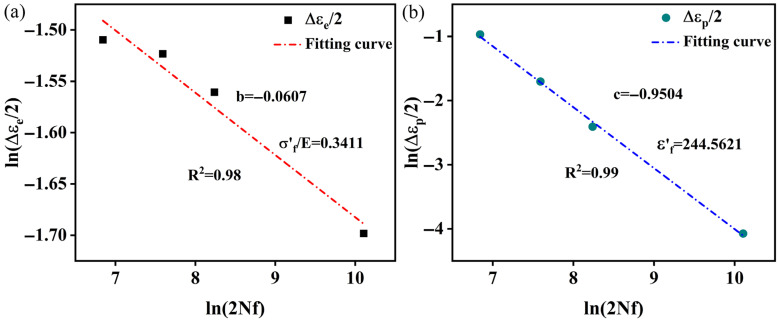
Coffin–Manson fits: (**a**) elastic strain amplitude $\left(\Delta \varepsilon_{e}/2\right)$–life $\left(2N_{f}\right)$ relation; (**b**) plastic strain amplitude $\left(\Delta \varepsilon_{p}/2\right)$–life $\left(2N_{f}\right)$ relation.

The plastic strain, elastic strain, and fatigue life of different strain amplitudes are illustrated in Table 2. The relationship between plastic strain and fatigue life, and the relationship between elastic strain and fatigue life, are fitted to determine the key parameters. The results are shown in Figure 15, and the coefficient of determination (R^2^) is 0.99. It can be determined that the values of b, $\sigma_{f}^{\prime }/E$, c, and $\varepsilon_{f}^{\prime }$ are −0.0607, 0.3411, −0.9504, and 244.5621, respectively. Thus, Equation (4) can be rewritten as Equation (7):

(7) $$\frac{\Delta \varepsilon_{t}}{2}=0.3411(2N_{f}{)}^{-0.0607}+244.5621(2N_{f}{)}^{-0.9504}$$

Based on the above analysis, the problem is reformulated as finding the root of a function, which is commonly solved using Newton–Raphson’s method. The objective function $f(N_{f})$ is defined as shown in Equation (8) [42]:

(8) $$f\left(N_{f}\right)=\frac{\sigma_{f}^{\prime }}{E}{\left(2N_{f}\right)}^{b}+\varepsilon_{f}^{\prime }{\left(2N_{f}\right)}^{c}-\frac{\Delta \varepsilon }{2}$$

**Figure 16:**
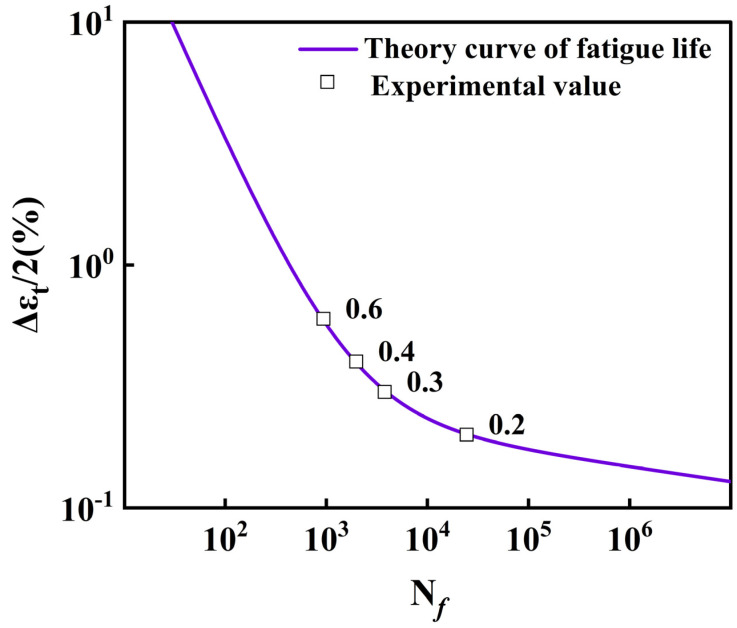
Coffin–Manson fatigue life prediction curve fitted to experimental data for 30Cr2Ni3MoWV steel at 700 °C.

The detailed implementation steps for fatigue life calculation are presented in Appendix A. The theoretical and experimental values of fatigue life are depicted in Figure 16. In this paper, δ is defined as the relative error of theoretical and experimental values of fatigue life.

(9) $$\delta =\left|\frac{N_{f}^{exp}-N_{f}^{num}}{N_{f}^{exp}}\right|\times 100\%$$

where $N_{f}^{exp}$ is the experimentally measured fatigue life, and $N_{f}^{num}$ is the numerically calculated fatigue life.

**Figure 17:**
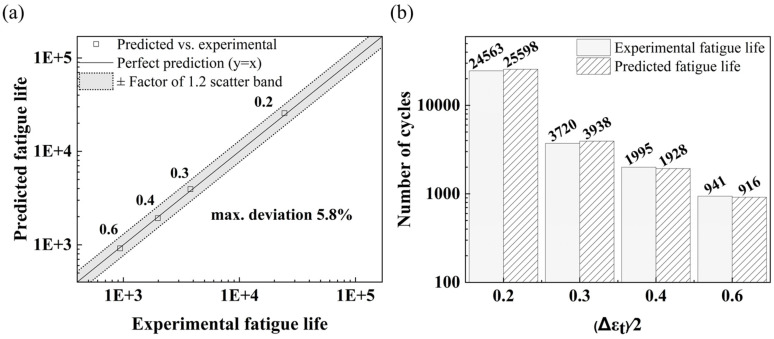
Experimental vs. predicted fatigue life: (**a**) predicted–experimental life correlation with a ±1.2 scatter band; (**b**) experimental and predicted lives at different strain amplitudes.

The Coffin–Manson relationship (Equation (8)) was fitted to the four measured fatigue data at strain amplitudes of 0.6%, 0.4%, 0.3%, and 0.2%. As shown in Figure 16, the fitted curve passes accurately through the experimental points. A direct comparison between experimental and predicted lives is provided in Figure 17, where all points fall well within a factor of 1.2 in a scatter band, with the maximum relative deviation being only 5.8% [43].

## 4. Conclusions

In this paper, the low-cycle fatigue performance of hot die steel under different strain amplitudes was studied, and the microstructure after fatigue fracture was further analyzed to reveal fatigue behavior. The main conclusions are as follows:

(1) The novel 30Cr2Ni3MoWV steel exhibits good high-temperature ductility at 700 °C, with a tensile strength of 460 MPa, 32% elongation, and 95% area reduction, demonstrating significant plastic deformation before fracture.
(2) Cyclic softening behavior intensifies with increasing strain amplitude, drastically reducing fatigue life. A defined stress-softening coefficient effectively quantifies this accelerated material degradation under higher strain conditions.
(3) Microstructural analysis confirms that coarser carbides (especially M_23_C_6_), increased dislocation density, and secondary cracking at carbide–matrix interfaces are the primary mechanisms driving accelerated softening and reduced fatigue life at high strain amplitudes.
(4) A Coffin–Manson relationship, based on the cyclic stress–strain response, accurately describes the low-cycle fatigue behavior of 30Cr2Ni3MoWV steel at 700 °C across the investigated strain amplitude range, achieving a maximum deviation of only 5.8% relative to the experimental data.

## Figures and Tables

**Table 1:** Chemical composition of the tested steel.

Element	C	Cr	Mo	W	Ni	V	Si	Mn	Fe
wt.%	0.25–0.32	2.5–3.2	1.6–2.5	0.2–1.0	0.5–1.5	0.2–1.0	≤0.5	≤0.5	Bal.

**Table 2:** Low-cycle fatigue test results of 30Cr2Ni3MoWV steel.

$(\Delta \varepsilon_{t}/2$)/%	$(\Delta \varepsilon_{e}/2$)/%	$(\Delta \varepsilon_{p}/2$)/%	$2N_{f}$
0.2	0.183	0.017	24,472
0.3	0.21	0.09	3786
0.4	0.218	0.182	1982
0.6	0.221	0.379	938

## Data Availability

The original contributions presented in this study are included in the article. Further inquiries can be directed to the corresponding author.

## Appendix A

All data processing, curve fitting, and genetic algorithm optimizations were performed using MATLAB R2024a (The MathWorks, Inc., Natick, MA, USA).

The detailed implementation of the steps of fatigue life calculation are as follows:

Given its efficiency (quadratic convergence rate) in solving single-variable nonlinear equations, the Newton–Raphson method is selected for this purpose. The iterative formula is as follows:

(A1) $$N_{f}^{\left(k+1\right)}=N_{f}^{\left(k\right)}-\frac{f\left(N_{f}^{\left(k\right)}\right)}{f^{\prime }\left(N_{f}^{\left(k\right)}\right)}$$

where the superscript k denotes the k-th iteration value. The first derivative $f^{\prime }\left(N_{f}\right)$ can be derived from the following equation:

(A2) $$f^{\prime }\left(N_{f}\right)=2b\frac{\sigma_{f}^{\prime }}{E}{\left(2N_{f}\right)}^{b-1}+2c\varepsilon_{f}^{\prime }{\left(2N_{f}\right)}^{c-1}$$

Input the necessary material parameters $\sigma_{f}^{\prime },\;\;b,\;\;\varepsilon_{f}^{\prime },\;\;c,\;\;E$ and the total strain amplitude $\frac{\Delta \varepsilon }{2}$. Choose an initial guess $N_{f}^{\left(0\right)}=10^{3}$ and set a convergence tolerance $\tau =10^{-6}$.

For each iteration until convergence is achieved, do the following:

Calculate the function value $f\left(N_{f}^{\left(k\right)}\right)\;$ using Equation (8);

Update the iteration value $N_{f}^{\left(k+1\right)}$ using Equation (A1);

Compute the derivative $f^{\prime }\left(N_{f}^{\left(k\right)}\right)$ using Equation (A2);

Check for convergence: $\left|f\left(N_{f}^{\left(k\right)}\right)\right|<\tau$ or $\left|N_{f}^{\left(k+1\right)}-N_{f}^{\left(k\right)}\right|/\left|N_{f}^{\left(k\right)}\right|<\tau$, terminate the iteration and output $N_{f}^{\left(k+1\right)}$ as the solution. The final value of $N_{f}$ represents the predicted fatigue life.
